# Development of a new hybrid model to enhance streamflow estimation using artificial neural network and reptile search algorithm

**DOI:** 10.1038/s41598-025-90550-x

**Published:** 2025-02-19

**Authors:** Mohammad Javad Bahmani, Zahra Kayhomayoon, Sami Ghordoyee Milan, Farhad Hassani, Mohammadreza Malekpoor, Ronny Berndtsson

**Affiliations:** 1 Department of Water Resources Engineering, Faculty of Civil Engineering, Azad University, Tehran, Iran; 2 Department of Geology, Payame Noor University, Tehran, Iran; 3 Department of Irrigation and Drainage Engineering, Aburaihan Campus, University of Tehran, Tehran, Iran; 4 The University of Texas at Arlington, Arlington, USA; 5 Department of Civil Engineering, Azarshahr Branch, Islamic Azad University, Azarshahr, Iran; 6 Division of Water Resources Engineering & Centre for Advanced Middle Eastern Studies, Lund University, Lund, Sweden

**Keywords:** Time series forecasting, Rainfall-runoff, Urmia Lake, Machine learning, Metaheuristic algorithms, Hydrology, Natural hazards

## Abstract

A new metaheuristic optimizer combined with artificial neural networks is proposed for streamflow prediction. Hence, the study aimed to forecast monthly streamflow of the main rivers in Urmia, Iran, by considering data shortage and using artificial neural network (ANN) models. By combining three variables: temperature, precipitation, and streamflow, we formulated five patterns, where 70% of the data were used for model training, and 30% for model testing. To improve the performance of ANN, we evaluated a new optimization algorithm, reptile search algorithm (RSA), and compared the results with combinations of ANN, particle swarm optimization algorithm (PSO), and whale optimization algorithm (WOA) models. The results of the ANN + RSA were promising at most stations and patterns. At Band station streamflow simulation testing gave RMSE, MAE, and NSE of 1.65, 1.21 MCM/month, and 0.80, respectively. At Babaroud station they were 4.01, 3.0 MCM/month and 0.68, respectively, at Nazlo station 5.62, 3.79 MCM/month, and 0.69, respectively, and at Tapik station 5.69, 3.82 MCM/month, and 0.59, respectively. However, the results of the ANN + PSO hybrid model were better than ANN + RSA. The impact of different parameters on the accuracy of streamflow prediction varied depending on model and streamflow station, indicating that the models do not perform consistently across different locations, times, and conditions. The inclusion of lagged monthly streamflow in the model was an influential input parameter. The results demonstrated that the new algorithm consistently improved predictions, enhancing the performance of traditional algorithms. The findings of this study highlight advantage of the ANN + RSA hybrid model for specific areas, suggesting its potential application in other similar hydrological problems for further validation.

## Introduction

Surface and groundwater resources are important water supplies^1^. Surface water resources are now more threatened than the groundwater resources due to easy exploitation and various pollution problems^2^. However, rivers play a key role in the hydrological ecosystem. The presence of rivers restores the environment and contributes to hydrological biodiversity. Therefore, it is essential to understand the effects of structural measures such as dams and reservoirs as well as non-structural strategies such as disaster prevention plans and flood control methods^3,4^. Meanwhile, various climatic factors as well as drought have a significant impact on the river flow, which can challenge the source of water supply. Therefore, simulating the river discharge and estimating it for the future period help managers and decision makers in planning. Also, it is important to have access to information about river flow to achieve optimal performance in river management and design flood warning systems^5^. Selecting suitable variables and the availability of data associated with the least uncertainty are important for forecasting accuracy. Mainly, two categories of information are required in river discharge estimation: physiographic and meteorological. Variables such as soil surface slope, vegetation cover, and river bed roughness affect the flow rate, and can be considered as a first category of variables. The existence and collection of such information at various locations of the river and at various time intervals are necessary to provide a conceptual model. However, due to the lack of sufficient funds and tools in different regions, this information is not always available. Especially in developing countries this may be a serious problem. Furthermore, variables such as temperature, evaporation, and precipitation impact river flow and make modeling of river flow a complex issue^5^. If both types of information are available, a conceptual model can be developed based on numerical models for simulation and prediction, which have high scenario adaptability. In some cases, when a large amount of information on rivers is not available, data-driven models can be used. Consequently, there are two main approaches in estimating river flow: conceptual and data-driven^6^. Conceptual approaches require mathematical tools, sufficient geophysical data, and adequate expertise and experience in the field^7^. In contrast, data–driven models do not require information about the physical nature of hydrological processes.

In case of sufficient time series information from a system, data–driven models can recognize hidden patterns. Machine learning models are of this category. In such models, by using meteorological data, it is possible to create an estimate of discharge. Due to the ease of use of these models, they have become popular in many fields in the last decade^8–13^. Statistical methods such as autoregressive integrated moving average (ARIMA) and seasonal autoregressive integrated moving average (SARIMA) were popular in the past decades as modeling tools for river flow and other hydrological phenomena^14–17^. However, machine learning models have gradually replaced these methods during the past two decades^18,19^. For instance, Huang et al.^20^ confirmed the accuracy of artificial neural networks (ANN) compared with statistical methods. However, ANN still suffers from weaknesses such as overfitting, falling into local minima, low convergence rate, and requiring a large amount of learning data^18^. This has led to the development of new optimization algorithms to enhance ANN results^21^. In recent years, different algorithms such as Genetic Algorithm (GA), Particle Swarm Optimization (PSO), Grey Wolf Optimizer (GWO), Differential Evolution (DE), Firefly Algorithm (FA), and Harris Hawks Optimization (HHO) have been developed to improve ANN^22^. Yaseen et al.^23^ used the FA optimization algorithm to improve the results obtained from the Adaptive Neuro-Fuzzy Inference System (ANFIS) for river flow forecasting. The findings showed that the hybrid algorithm ANFIS + FA is more accurate than ANFIS. Whale Optimization Algorithm (WOA) has been shown to enhance the results of non-hybrid models and successfully optimized models such as ANFIS, ANN, and Support Vector Regression (SVR)^24–27^. The PSO is inspired by nature and creates a random population with each particle presenting a solution and eventually selects the best one. Ghorbani et al.^28^ confirmed the efficiency of this algorithm in optimizing the results of ANN.

The Reptile Search Optimization Algorithm is one of the latest optimization algorithms based on the model from nature, proposed in 2022. This algorithm has been used in various optimization fields such as Churn Prediction^29^, Selective Harmonic Elimination Technique in Packed E–Cell (PEC–9) Inverter^30^. It has, however, rarely been combined with neural network and ANFI models and for forecasting problems. In this context, its application in prediction of COVID–19 cases in Europe with ANN + RSA by Manohar et al.^31^ and groundwater levels under representative concentration pathway scenarios using ANN + WEA by Ehteram et al.^32^ can be mentioned. However, it so far not been used extensively in hydrological forecasting. Other algorithms such as particle swarm and whale hunting have been used in hydrological forecasting of runoff, groundwater level, soil moisture^33^, and the use of the GWO and neural networks in predicting monthly river flow, predict crop yield, and wilting point^34^. The use of RSA and PSO algorithms for predicting groundwater levels under the influence of climate change was done^32^.

However, the comparison of hybrid models that utilize new evolutionary algorithms for ANN training is less common. Lake Urmia is one of the most important lakes in Iran, whose area has been decreasing severely over the last two decades. The most important rivers that flow into this lake are Nazlo Chay, Shahr Chay, Roze Chay, and Barandouz Chay. To improve the performance of the ANN, optimization algorithms, including WOA, RSA, and PSO were used to tune the parameters of the machine learning. The performance of the hybrid models was compared with that of ANN and hydrological models. The streamflow was estimated for the four main rivers at different discharge stations (Nazlo, Tapik, Band, and Babaroud stations) using available meteorological data. Various input scenarios for the models with a combination of input variables were assumed to consider data scarcity. The most suitable model and scenario for river flow forecasting was identified based on error evaluation criteria.

## Materials and methods

**Fig. 1:**
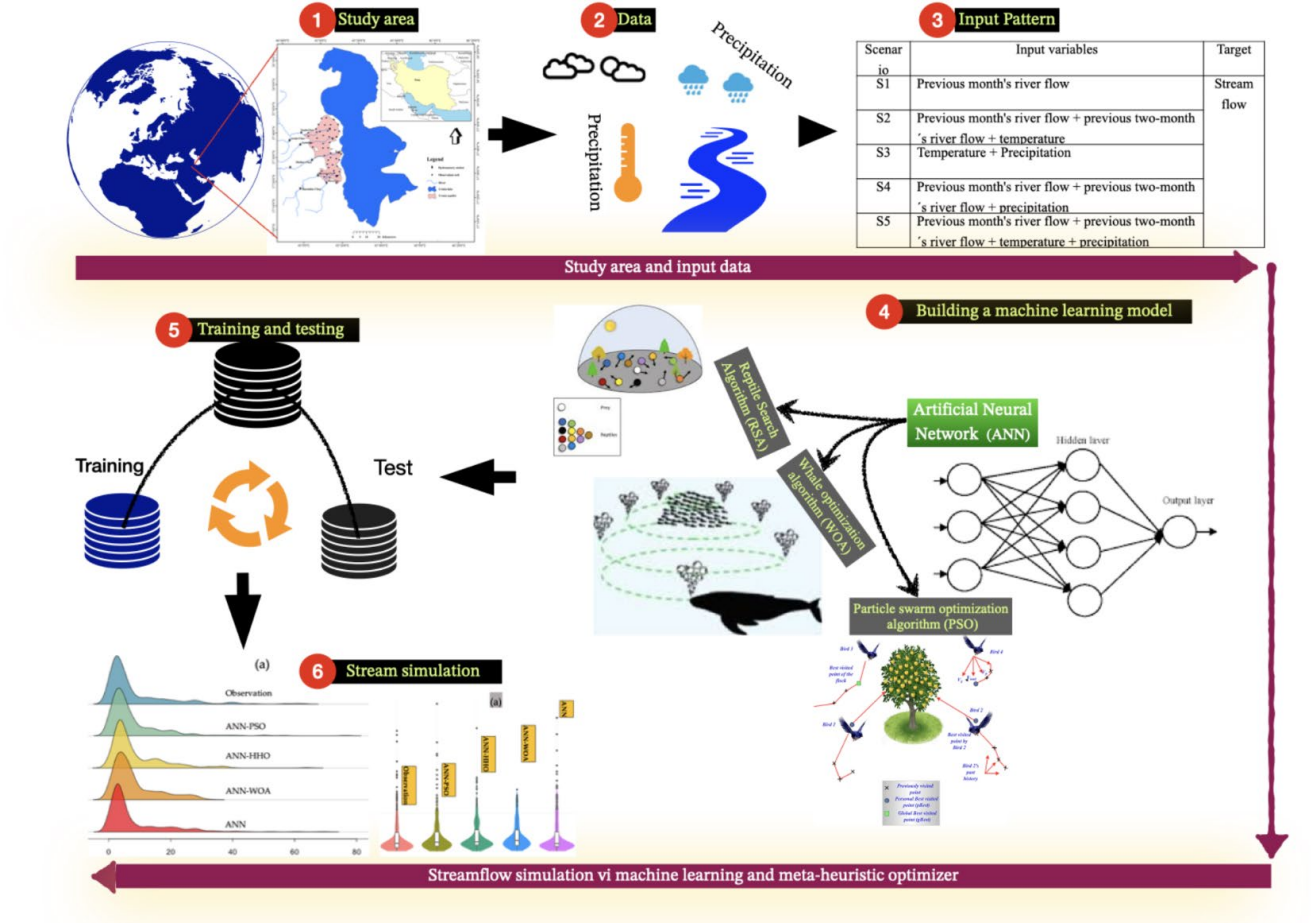
Schematic flowchart of river flow forecasting methodology used in the present study.

We studied ways to improve flow forecasting using data-driven methods according to the methodological flowchart presented in Fig. 1. Thus, we tested a minimum of variables to evaluate effects on the streamflow modeling. Monthly observations of temperature and precipitation were used to forecast river flow. For this purpose, the main rivers that flow into Lake Urmia were selected, and in each river, different station data were considered for simulating the flow rate. At first, the ANN was used to predict the river flow. Then, metaheuristic algorithms, i.e., PSO, WOA, and RSA, were used to improve the hyperparameters of the ANN.

Training and testing data were selected randomly with a ratio of 70:30. Several input scenarios were defined based on combinations of input data. The results of machine learning models were compared to results of a conceptual hydrological model using precipitation and temperature data. The comparison of the results of the models as well as the input scenarios was based on error evaluation criteria and various graphic evaluation diagrams. These comparisons were made in order to select the most appropriate model and input scenario.

### Study area

**Fig. 2:**
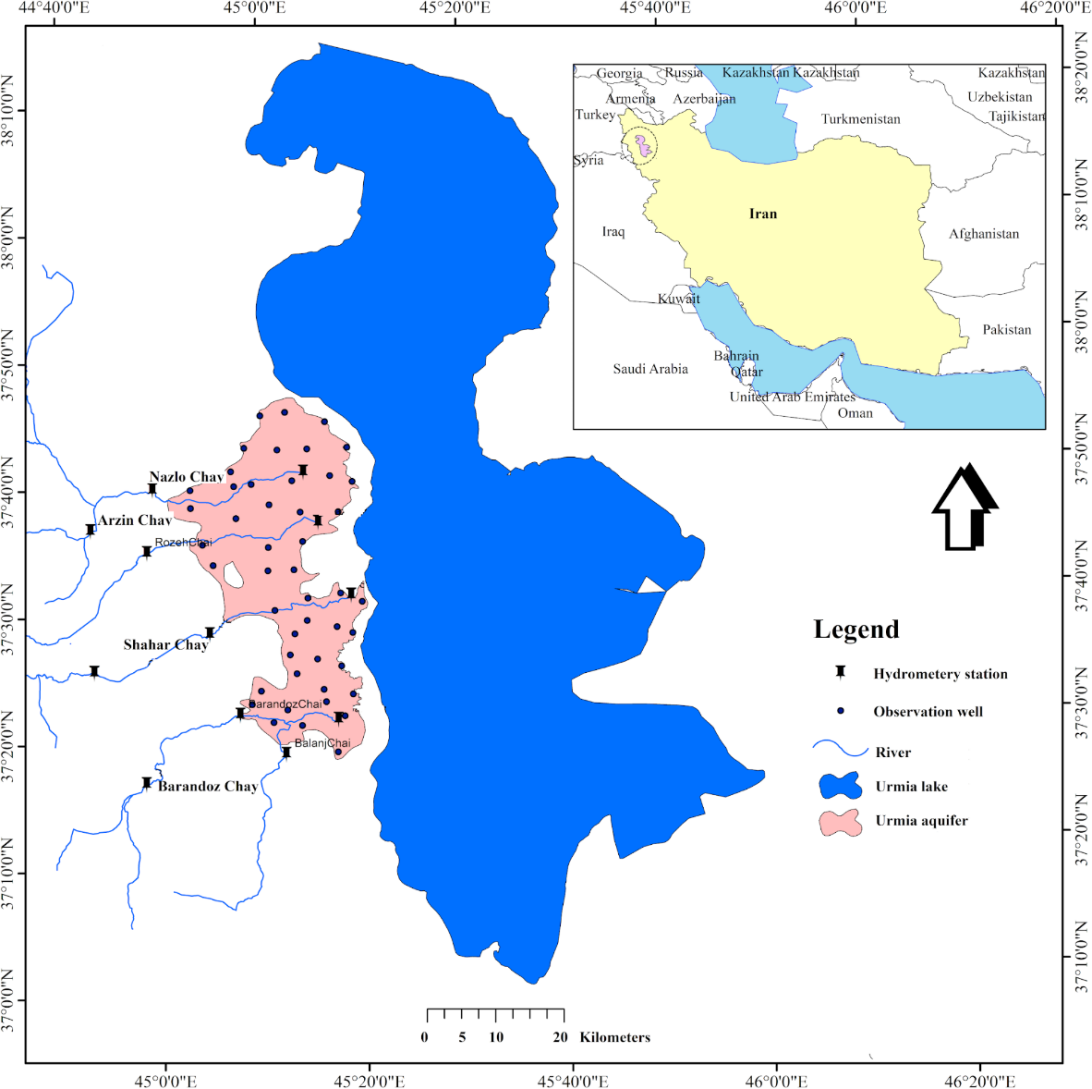
Location of Lake Urmia in Iran with its four main river inflow.

Urmia Lake basin is one of the main water basins of Iran. This basin consists of different sub-basins, of which the Urmia sub-basin is the most important. Urmia sub-basin is located in the northwest of Iran, which has temperate and humid weather. The direction of water flow is from east to west, which starts from the heights and finally discharges into Lake Urmia. The average temperature of the area is about 20.0 °C, with an average annual rainfall of about 300.0 mm. The sub-basin has large amounts of water resources and fertile soil and high–quality agriculture. It has, however, been drying up in recent years due to many reasons, including urban and agricultural development, lack of allocation of water rights, climate change, and drought. Due to the high salinity of the lake and in case of drying up, it can cause dangerous salt dust in the region, and therefore, its revival is on the agenda of the government. Much research has been performed to suggest solutions for its revival and investigate the causes of its dryness. Forecasting the flow of rivers in the region can be a step in this procedure. The main rivers of the sub-basin include Nazlo Chay, Shahr Chay, Roze Chay, and Barandouz Chay, which were evaluated in this study (Fig. 2).

### Data used

**Fig. 3:**
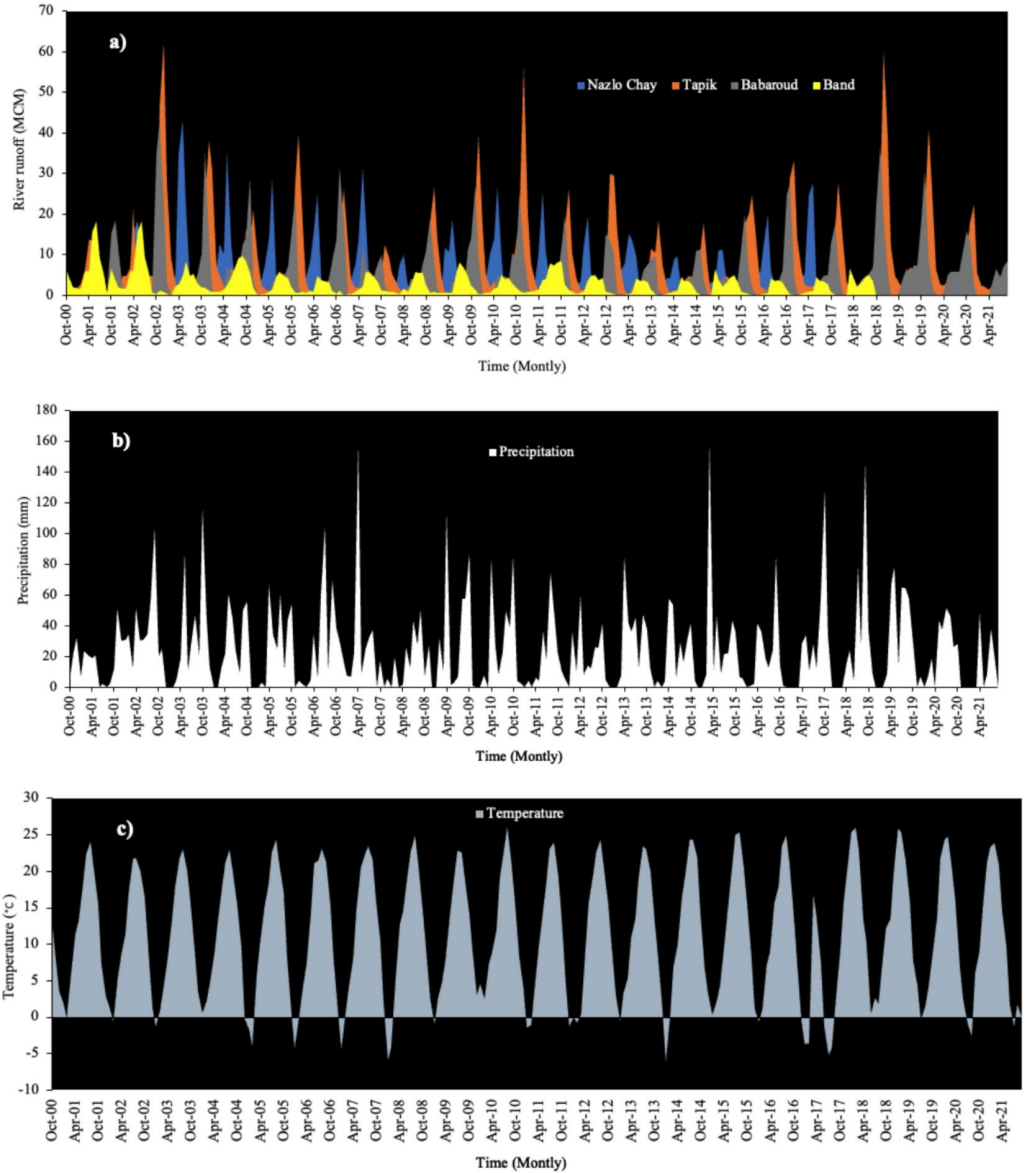
Time series of streamflow, precipitation, and temperature for the 2001‒2021 period: (**a**) steamflow, (**b**) precipitation, (**c**) temperature.

Monthly temperature, precipitation, and runoff were used to evaluate the performance of models in river flow forecasting. The monthly data were collected from 2001 to 2021 (Fig. 3). Average monthly streamflow was higher at the beginning of the study period compared to the end of the period. The average river flow rate during the entire examination period was about 5.40 MCM/month. Changes in precipitation that occurred during the experimental period were to some extent reflected the changes in river flow rates (Fig. 3b). Precipitation decreased during the observation period. Average precipitation and standard deviation were 18.8 and 23.0 (mm/month), respectively. Monthly temperature fluctuated between 0.0 and 23.0 °C. Maximum and minimum temperatures occurred in summer and winter, respectively. The average temperature was 11.6 °C and the standard deviation was 8.8 °C (Iran Water Resources Management Company, 2017).

To evaluate the models, five scenarios were considered depending on the combination of input variables (Table 1). The first input pattern included the flow of the previous month. The second input pattern included the previous month and previous two-month flow and temperature. Temperature and precipitation constituted the third scenario. The fourth scenario included the second scenario and precipitation. Finally, the fifth scenario was the combination of all input variables. Each scenario was simulated by using the models and the results were analyzed.

**Table 1:** Combinations of input variables for streamflow simulations.

Pattern	Input variables	Target
S1	Previous month’s streamflow	Monthly streamflow
S2	Previous month’s streamflow + previous two–month´s streamflow + temperature	
S3	Temperature + Precipitation	
S4	Previous month’s streamflow + previous two–month´s streamflow + precipitation	
S5	Previous month’s streamflow + previous two–month´s streamflow + temperature + precipitation	

### Artificial neural networks

ANNs are parallel distributed information processing systems that perform similarly to human neural networks^35^. An artificial neural network consists of one or more hidden layers, an input layer, and an output layer. Neurons transfer information from one layer to another using various functions^36^. The weights and biases in each layer of the neural network are determined by traditional algorithms. In this research, a hidden layer with 8 neurons was utilized. These neurons form the transfer functions in the hidden and output layers, respectively, using hyperbolic tangent sigmoid (TANSIG) and pureline functions. The Levenberg–Marquardt algorithm was employed to train the neural network structure. Firstly, the scenarios with different variables are given to the model as input. After selecting the model inputs, the number of hidden and output layers and their size are first determined. The network evaluation criterion is then selected to calculate the error between the observed and predicted values of the networks. The ANN determines the weights and bias of the neurons by different algorithms based on training data^37^. This step is repeated until the error between the observed and predicted values becomes lower than a threshold. Therefore, the objective function in this structure is to minimize the difference between the observed and predicted values. Finally, the river flow is predicted by the trained model. Along with the ANN model, several metaheuristic algorithms (RSA, PSO, WOA) were used to improve the ANN performance. Each of these algorithms has its unique features that can significantly improve the traditional ANN model.

### Metaheuristic optimizer

#### Particle swarm optimization algorithm

**Fig. 4:**
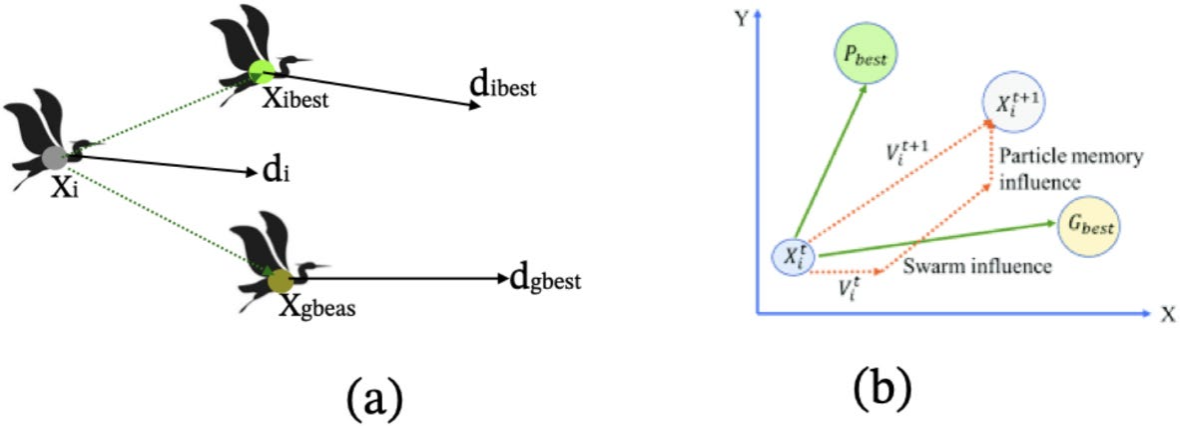
(**a**) Bird position in nature, (**b**) in the Cartesian coordinate system.

The PSO algorithm, introduced by Kennedy and Eberhart (1995), is inspired by nature^38^. It begins by creating a random population, where each member is a unique set of decision variables whose optimal values need to be determined. Each member is represented as a vector within the problem-solving space. Besides the position vector, the algorithm also uses a velocity vector, which helps adjust the positions of the population in the search space. The velocity is composed of two vectors: *p*, representing the best position a particle has ever achieved, and *p*_*g*_, representing the best position achieved by any particle in its vicinity. Each particle provides a solution in every iteration of the algorithm (Fig. 4a,b). When searching in a *d*–dimensional space, the position of particle *i* is depicted as a *D*–dimensional vector called *X*_*i*_ = (*X*_*i*1_, *X*_*i*2_, …, *X*_*i*D_). The velocity of each particle is shown by a *D*–dimensional velocity vector called *V*_*i*_ = (*V*_*i*1_, *V*_*i*2_, …, *V*_*i*D_). Finally, the population moves to the optimum point using (Eqs. 1 and 2):

1 $${V}_{id}^{n+1}=X(\omega .{v}_{id}^{n}+{c}_{1}{r}_{1}^{n}\left({p}_{id}^{n}-{x}_{id}^{n}\right)+{c}_{2}{r}_{id}^{n}\left({p}_{pg}^{n}-{x}_{id}^{n}\right))$$

2 $${x}_{id}^{n+1}={x}_{id}^{n}+{v}_{id}^{n+1}$$

where *ω* is the shrinkage factor used for convergence rate determination, *r*_1_ and *r*_2_ are random numbers between 0 and 1 with uniform distribution, *N* is the number of iterations, *c*_1_ is the best solution obtained by a particle, and *c*_2_ is the best solution identified by the whole population. For further reading on WOA please see^38^.

#### Whale optimization algorithm

**Fig. 5:**
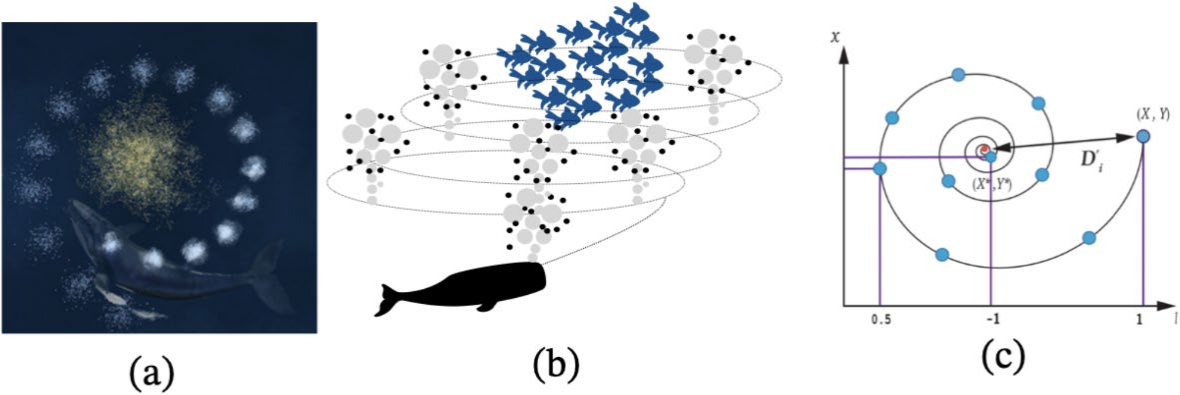
The position of the whale and prey, (**a**) in nature, (**b**) in a simulated state, and (**c**) in a mathematical coordinate system.

The WOA, introduced by Mirjalili and Lewis (2016), is based on the bubblenet hunting behavior of humpback whales^39^. Whales can identify the location of their prey and encircle it (Fig. 5a,b). However, since the optimal position in the search space is not known, the algorithm assumes that the best current solution is the closest prey (Fig. 5c). Once this point is identified, the search for other optimal points and the updating of positions proceeds, which is indicated by (Eqs. 3 and 4):

3 $$D=\left|\overrightarrow{c.} \overrightarrow{{X}_{t}^{*}}-\overrightarrow{{X}_{t}}\right|$$

4 $${\overrightarrow{X}}_{t+1}={\overrightarrow{X}}_{t}^{*}-\overrightarrow{A}.\overrightarrow{D}$$

where *t* is the current iterator, *C* and *A* are the coefficient vectors, X^*^ is the best position vector so far, and *X* is the position vector. The vectors *A* and *C* are calculated as (Eqs. 5 and 6):

5 $${\overrightarrow{X}}_{t+1}={\overrightarrow{X}}_{t}^{*}-\overrightarrow{A}.\overrightarrow{D}$$

6 $$\overrightarrow{C}=2.\overrightarrow{r}$$

where *a* is a vector in both exploration and exploitation phases that is reduced from 2.0 to 0.0 per repetition, and *r* is a random vector in the range [0, 1].

#### Reptile search algorithm

**Fig. 6:**
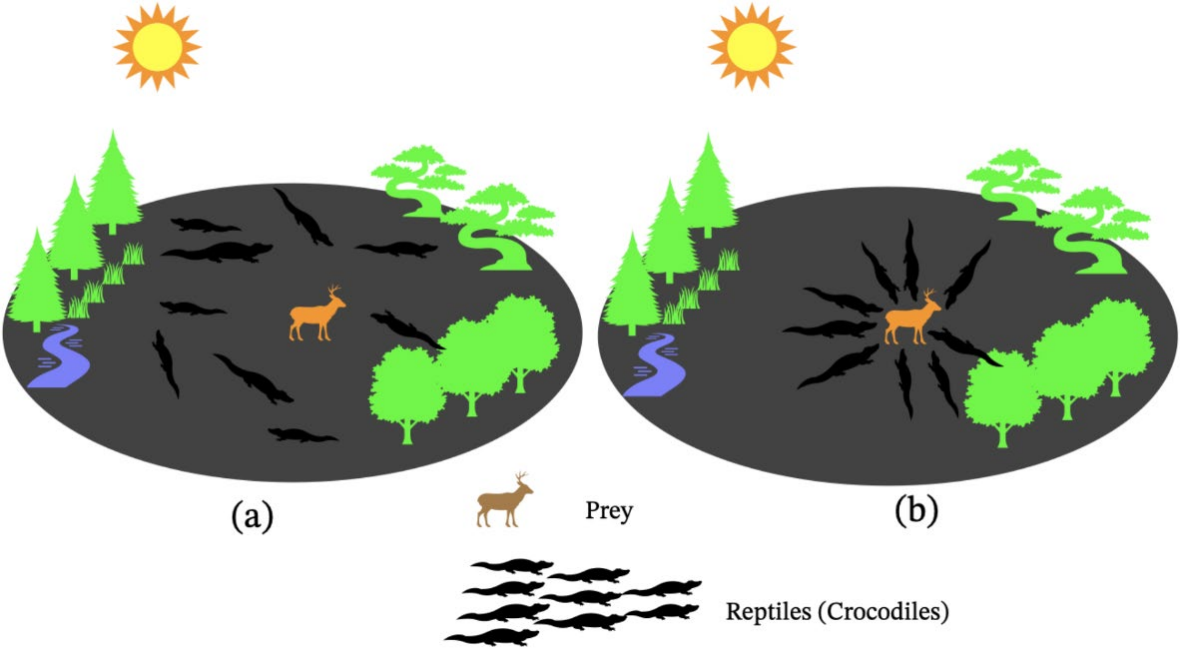
Encircling the prey; (**a**) encircling the prey, (**b**) attacking the prey.

The RSA is a metaheuristic algorithm inspired by the predatory strategy of crocodiles to search for food. Crocodile behavior was divided into two categories including high walking and belly walking in terms of exploration strategy^25^. The total number of repetitions was divided into four parts based on these methods. In the exploration strategy, two conditions for high walking ($t\le \frac{T}{4.0}$) and belly walking ($t>\frac{T}{4.0} and\, t\le \frac{2T}{4.0}$) must be met (Fig. 6a,b). The method of RSA algorithm can be summarized as follows:

The RSA algorithm is implemented by starting with solutions chosen at random and generating them as (Eq. 7):

7 $${y}_{(i,j)}=rand\times \left(UB-LB\right)+LB, j=\text{1,2},\dots ,n$$

In the encircling phase, updating the solution’s position is specified as (Eq. 8):

8 $$\begin{aligned} y_{{\left( {i,j} \right)}} \left( {t + 1} \right) & = \{ Best_{j1} \left( t \right) \times h_{1} \left( t \right) \times \beta_{1} - R_{{1\left( {i,j} \right)}} \times rand, \\ t & \le \frac{T}{4} Best_{j1} \left( t \right) \times x_{{\left( {r,j} \right)}} \times ES_{1} \left( t \right) - R_{{1\left( {i,j} \right)}} \times rand, t > \frac{T}{4} \,and \,t \\ &\le \frac{2T}{4} \\ \end{aligned}$$

${Best}_{j1}\left(t\right)$ is the best previous solution, *rand* is a random number between 0.0 and 1.0^39^. In addition, *b*_*1*_ is a critical parameter that affects the heuristic performance, while *t* and *T* reflect the current and total number of iterations. *ES*(*t*) is the random value between –2 and 2 in all iterations evaluated using Eq. (9), and *x*(*r*_*1*_*,j*) is the arbitrary position of solution *i*.

**Fig. 7:**
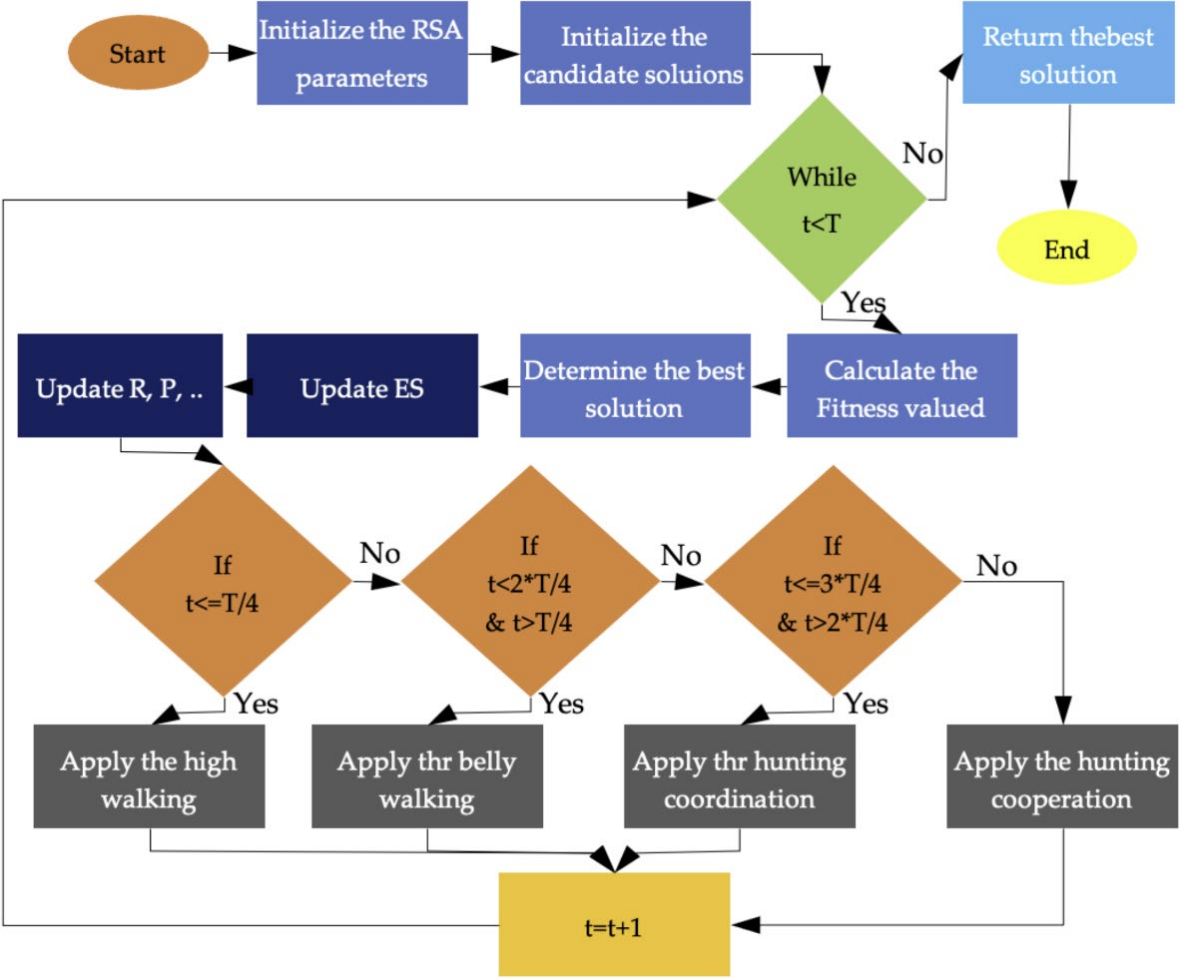
Flowchart of the RSA algorithm optimization.

*R*_1(*i*,*j*)_ is a diminished search area that is computed using Eq. (11), *r*_1_ is the random choice lying in [1*N*], where *N* is the total number of solutions and Eq. (10) is used to calculate *h*_1(*i*,*j*)_. It defines the hunting operator to the *j*th position of *i*th solution^39^ (Fig. 7).

9 $${ES}_{1}(t)={2\times r}_{3}\times \left(1-\frac{1}{T}\right)$$

10 $${{h}_{1(i,j)}=Best}_{j1}\left(t\right)\times {h}_{1}\left(t\right)\times {P}_{1}-{R}_{1\left(i,j\right)}$$

11 $${R}_{1\left(i,j\right)}=\frac{{Best}_{j1}\left(t\right)-{x}_{\left({r}_{2},j\right)}}{{Best}_{j1}\left(t\right)+\epsilon }$$

Here $\epsilon$ represents a small value, *r*_2_ belongs to [1, *N*], and *r*_3_ signifies the arbitrary value in [*–*1, 1]. In the RSA algorithm’s exploitation phase, we utilized Eq. (12) to calculate the new solution^39^.

12 $$\begin{aligned} y_{{\left( {i,j} \right)}} \left( {t + 1} \right) & = \{ Best_{j1} \left( t \right) \times h_{1} \left( t \right) \times P_{{1\left( {i,j} \right)}} \times rand, \\ t & > \frac{2T}{4} t \le \frac{3T}{4} Best_{j1} \left( t \right){-}h_{{1\left( {i,j} \right)}} \left( t \right) \times \epsilon - R_{{1\left( {i,j} \right)}} \times rand, t & \frac{3T}{4} \;and\; t\\ &\le T \\ \end{aligned}$$

**Fig. 8:**
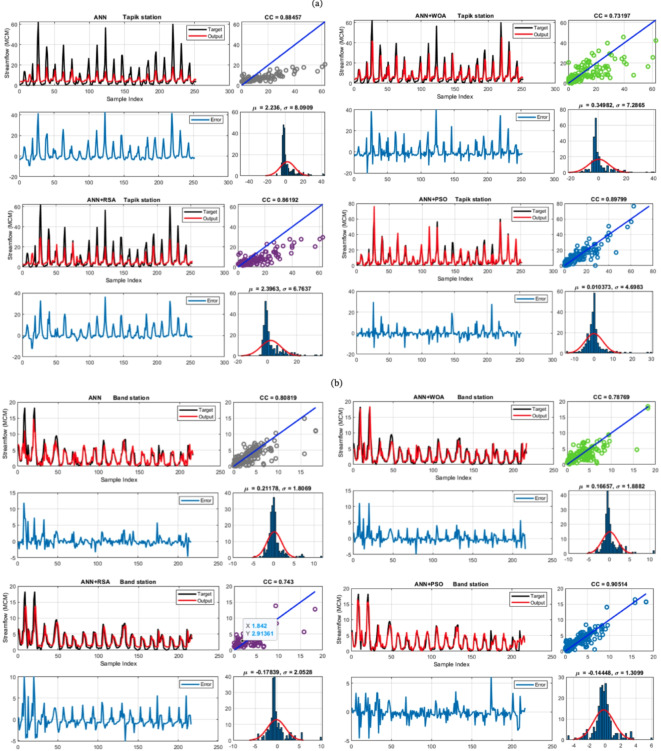

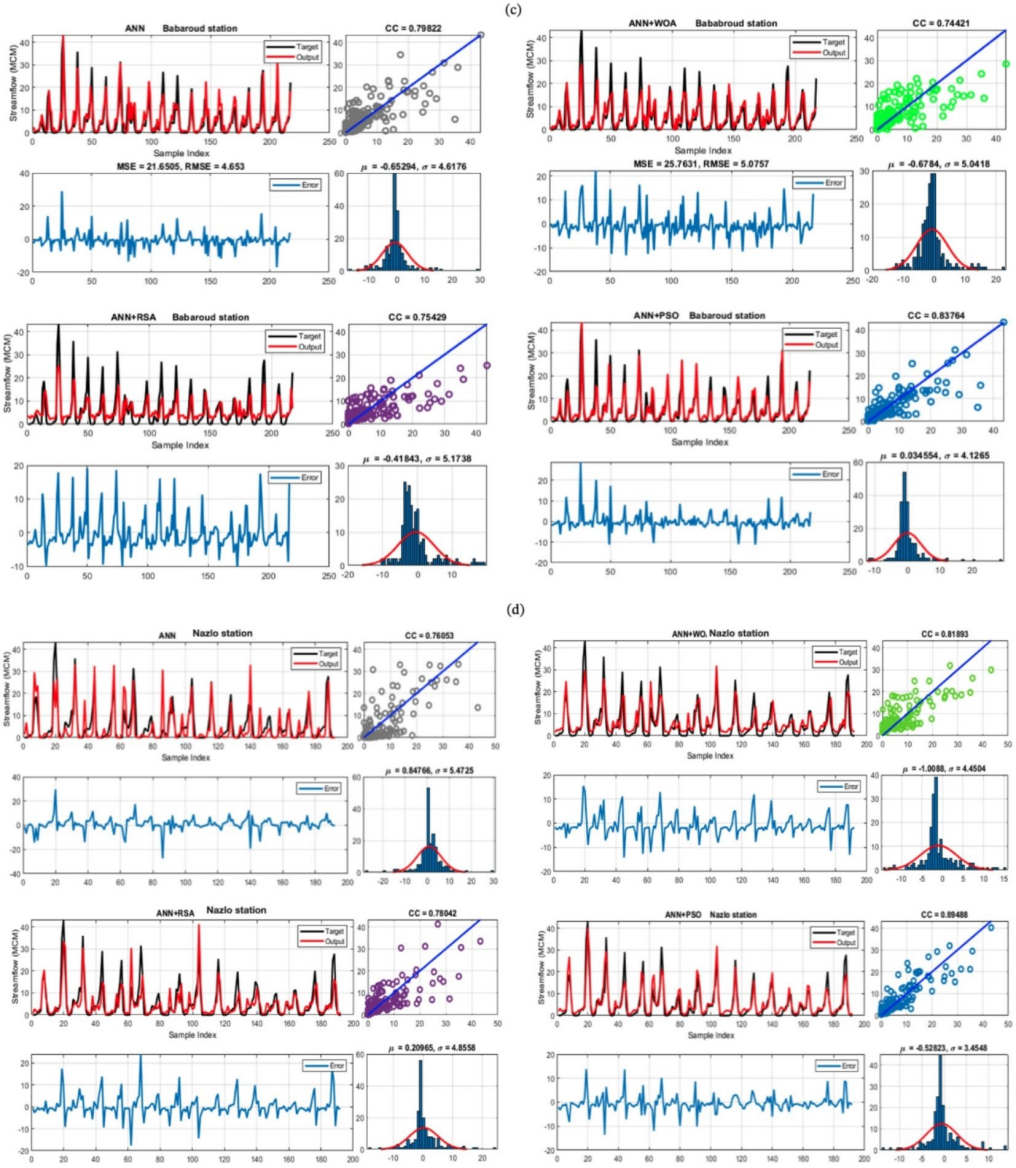
Time series of calculated and observed streamflow and error charts of each model; All datasets, (**a**) Tapik station, (**b**) Band station, (**c**) Babaroud station, and (**d**) Nazlo station.

*P*_1(*i*,*j*)_ (*t*) is defined as the discrepancy of percentage determined by Eq. (13) among *j*th place of the best one and *j*th place of the current one (Fig. 8).

13 $${P}_{1\left(i,j\right)}\left(t\right)=\alpha +\frac{{x}_{(i,j)}-{M}_{1}({x}_{i})}{{Best}_{j1}\left(t\right)\times ({UB}_{j}-{LB}_{j})+\epsilon }$$

where $\alpha$, another parameter with fixed value 0.1, is used to restraint the exploration precision and *M*_1_(*x*_*i*_) is computed by Eq. (14) as:

14 $$M_{1} \left( {x_{i} } \right) = \frac{1}{n}\sum\limits_{{j = 1}}^{n} {x_{{(i,j)}} }$$

### Performance evaluation metrics

To evaluate the performance of the machine learning models, several error evaluation criteria were used. The dataset was randomly divided into two groups, with 70% of data for model training and 30% of the data for validation. Root mean square error (RMSE) (Eq. 15), Nash Sutcliffe Index (NSE) (Eq. 16), and mean absolute error (MAE) (Eq. 17) were considered for the evaluation based on:

15 $$Root \;mean \,square \;error = \sqrt {\frac{{\mathop \sum \nolimits_{i = 1}^{n} \left( {streamflow_{i}^{{\left( {obs} \right)}} - streamflow_{i}^{{\left( {est} \right)}} } \right)^{2} }}{number\; of\; samples}}$$

16 $$Nash \;Sutcliffe \;Index = 1 - \frac{{\mathop \sum \nolimits_{i = 1}^{n} \left( {streamflow_{i}^{{\left( {obs} \right)}} - streamflow_{i}^{{\left( {est} \right)}} } \right)^{2} }}{{\mathop \sum \nolimits_{i = 1}^{n} \left( {streamflow_{i}^{est} - \overline{{streamflow^{obs} }} } \right)^{2} }}$$

17 $$Mean\; absolute\; error = \frac{{\mathop \sum \nolimits_{i = 1}^{n} \left| {streamflow_{i}^{{\left( {obs} \right)}} - streamflow_{i}^{{\left( {est} \right)}} } \right|}}{number\; of\; samples}$$

where *i* represents the streamflow in *i*th step. $\overline{streamflo{w}^{obs}}$ represent the average of the observed steamflow. The lower RMSE and MAE and higher NSE indicate better model performance.

## Results

The PSO, RSA, and WOA algorithms used 3000 iterations because there was no improvement in simulation after this. Among the populations considered for each algorithm, 35.0, 30.0, and 30.0 population sizes were obtained for the RSA, PSO, and WOA algorithms, respectively. The alpha and beta values in the RSA algorithm were considered to be 0.1 and 0.05, respectively.

As Table 2 shows, we calculated the error assessment criteria for all models and the related input scenarios involved. Results of the hybrid models were similar to the estimation of discharge at the Tapik station with acceptable accuracy. According to Table 2 and in the Tapik station, the ANN + RSA (RMSE = 5.69 MCM/month, MAE = 3.82 MCM/month, and NSE = 0.59d for test data) and ANN + PSO (RMSE = 6.28 MCM/month, MAE = 3.42 MCM/month, and NSE = 0.75 for test data) models performed better than the ANN + WOA (RMSE = 7.46 MCM/month, MAE = 4.55 MCM/month, and NSE = 0.73 for test data) hybrid model. Among the scenarios, S4 and S5 performed better for most stations and models. The NSE values in the test data section are in the appropriate range in most cases, the highest value of which reaches 0.88. This value is related to the estimate of discharge in the Tapik station and in the ANN + RSA model, in which S1 had the most suitable performance among the scenarios. In the Babaroud station and ANN + WOA model results, the lowest NSE values were always observed. In this station, the S4 scenario, which was the selected scenario in ANN + WOA, reached the NSE value of 0.55 in the test data.

**Table 2:** Error assessment criteria of the forecasting models in the Tapik, Band, Babaroud, and Nazlo stations.

Station	Model	Scenario	RMSE MCM/month		MAE MCM/month		NSE	
			Training	Test	Training	Test	Training	Test
Tapik	ANN	S1	6.44	8.77	3.94	5.15	0.78	0.49
		S2	3.98	7.89	2.23	4.37	0.92	0.42
		S3	7.41	8.52	3.50	4.53	0.82	0.57
		S4	6.48	5.85	3.58	3.46	0.66	0.75
		**S5**	**5.72**	**5.50**	**4.13**	**4.84**	**0.58**	**0.53**
	ANN + RSA	***S1***	**6.54**	**5.69**	**4.38**	**3.82**	**0.57**	**0.59**
		S2	6.07	6.16	4.20	4.20	0.64	0.35
		S3	6.52	6.22	4.80	4.26	0.53	0.54
		S4	5.97	6.64	4.25	4.48	0.58	0.55
		***S5***	6.23	6.96	4.43	4.76	0.51	0.56
	ANN + PSO	S1	7.22	8.30	4.57	4.70	0.66	0.64
		S2	4.86	6.58	2.89	3.48	0.88	0.63
		S3	7.33	6.95	4.35	4.67	0.75	0.48
		**S4**	**3.80**	**6.28**	**2.35**	**3.42**	**0.92**	**0.75**
		S5	3.28	7.36	1.89	4.21	0.94	0.66
	ANN + WOA	S1	8.24	9.11	5.01	6.01	0.62	0.57
		**S2**	**5.42**	**7.46**	**3.60**	**4.55**	**0.80**	**0.73**
		S3	7.83	7.81	5.01	4.84	0.62	0.63
		S4	7.34	7.78	4.61	4.44	0.69	0.70
		S5	5.88	8.35	4.23	4.76	0.79	0.61
Band	ANA	S1	1.54	1.55	3.23	1.08	0.87	0.82
		**S2**	**1.07**	**2.13**	**2.14**	**1.38**	**0.92**	**0.77**
		S3	1.62	3.67	2.80	2.39	0.83	0.37
		S4	1.18	2.61	2.32	1.77	0.92	0.59
		S5	1.25	2.28	2.05	1.30	0.90	0.75
	ANN + RSA	**S1**	**2.20**	**1.65**	**1.51**	**1.21**	**0.74**	**0.80**
		S2	2.08	2.11	1.48	1.30	0.77	0.66
		S3	2.71	2.80	1.71	2.00	0.58	0.51
		S4	2.21	2.03	1.67	1.63	0.75	0.64
		S5	2.06	2.06	1.29	1.32	0.74	0.77
	ANN-PSO	S1	1.46	1.91	3.14	1.32	0.86	0.83
		**S2**	**1.17**	**1.60**	**2.25**	**1.11**	**0.93**	**0.83**
		S3	2.13	2.59	3.80	1.81	0.76	0.53
		S4	1.37	2.41	2.82	1.38	0.89	0.69
		S5	1.23	1.83	2.32	1.16	0.92	0.75
	ANN + WOA	S1	2.49	2.44	4.96	1.61	0.65	0.65
		S2	2.11	2.70	4.40	1.77	0.73	0.59
		S3	2.73	2.46	5.40	1.55	0.59	0.62
		S4	1.89	3.13	3.86	1.45	0.78	0.49
		**S5**	**1.46**	**2.65**	**3.01**	**1.56**	**0.87**	**0.65**
Babaroud	ANN	S1	5.44	6.07	3.24	3.53	0.68	0.50
		S2	3.45	6.85	1.51	3.30	0.85	0.45
		S3	6.01	4.57	3.88	7.70	0.58	0.14
		S4	4.41	5.88	2.40	3.19	0.81	0.46
		**S5**	**3.23**	**5.56**	**1.36**	**2.88**	**0.86**	**0.74**
	ANN + RSA	S1	5.87	7.02	3.83	4.00	0.63	0.44
		S2	6.08	7.30	4.45	5.24	0.59	0.47
		S3	7.14	6.58	5.29	4.64	0.36	0.42
		**S4**	**6.55**	**4.01**	**4.15**	**3.00**	**0.63**	**0.68**
		**S5**	5.91	7.81	4.19	5.38	0.56	0.55
	ANN + PSO	S1	5.53	5.99	3.26	3.41	0.66	0.56
		S2	2.57	6.18	1.68	3.40	0.92	0.59
		S3	5.82	7.75	4.00	5.17	0.61	0.36
		S4	3.93	5.67	2.16	3.96	0.83	0.63
		**S5**	**2.65**	**5.84**	**1.79**	**3.30**	**0.91**	**0.71**
	ANN + WOA	S1	6.10	6.07	3.87	4.08	0.57	0.60
		S2	6.65	8.36	4.22	5.13	0.51	0.19
		S3	8.22	5.85	5.51	4.27	0.41	0.33
		**S4**	**5.37**	**4.90**	**3.87**	**2.41**	**0.64**	**0.55**
		S5	5.61	6.26	3.22	3.92	0.61	0.60
Nazlo	ANN	**S1**	**5.83**	**6.13**	**3.90**	**4.12**	**0.59**	**0.52**
		S2	6.50	7.29	4.53	5.47	0.33	-0.76
		S3	6.70	7.54	4.89	0.25	0.26	-0.017
		S4	6.42	9.60	4.46	7.35	0.21	-0.80
		S5	6.37	6.81	4.65	5.15	0.23	0.04
	ANN + RSA	S1	6.16	5.81	3.93	3.92	0.59	0.58
		S2	5.86	5.72	4.03	4.46	0.64	0.53
		S3	6.57	6.80	4.74	5.13	0.52	0.45
		S4	5.00	7.37	3.00	3.52	0.74	0.22
		**S5**	**5.51**	**5.62**	**3.47**	**3.79**	**0.64**	**0.69**
	ANN + PSO	S1	3.33	5.40	1.75	2.53	0.71	0.70
		S2	4.26	4.25	2.12	2.16	0.71	0.62
		S3	4.66	4.94	3.45	3.23	0.68	0.49
		**S4**	**3.95**	**4.20**	**2.18**	**2.71**	**0.66**	**0.69**
		S5	3.96	5.84	2.23	3.89	0.48	0.47
	ANN + WOA	S1	4.60	5.31	2.80	0.61	0.81	0.67
		**S2**	**6.40**	**5.41**	**4.55**	**3.53**	**0.45**	**0.55**
		S3	4.82	6.14	2.63	4.84	0.60	0.43
		S4	4.36	5.89	2.61	3.40	0.62	0.40
		S5	6.12	6.75	4.19	5.26	0.56	0.38

Selected patterns in each station are highlighted in bold and bolditalic.

However, the results of ANN + RSA were promising in most of the patterns. Among the patterns in this model, the S1 pattern provided better results at the Band and Tapik stations. At the Band station, the values of RMSE = 1.65 MCM/month, MAE = 1.21 MCM/month, NSE = 0.80 were obtained for the test data, which were respectively equal to 2.20 MCM/month, 1.51 MCM/month, and 0.74 for the training data. The fourth pattern was better than the other patterns in the Babaroud station with the values of RMSE = 4.01 MCM/month, MAE = 3.00 MCM/month, NSE = 0.68 for the test data, which were respectively equal to 6.55 MCM/month, 4.15 MCM/month, and 0.63 for the training data, confirming the results.

The error assessment criteria for the Nazlo station were low for the ANN model in all considered scenarios. Using the variables in Scenario S1 lead to optimum results for this model. Thus, the NSE, and RMSE for test data were 0.52, and 6.13 MCM/month respectively. Using the three evolutionary algorithms WOA, PSO, and RSA improved the accuracy of the ANN model in forecasting training and test data. Among the three evolutionary algorithms, the ANN + PSO hybrid model obtained the best results under Scenario S4 (NSE = 0.69, RMSE = 4.20 MCM/month for test data). The results of the scenarios showed that with only a 1-month or 2-month delay for the runoff, it is not possible to achieve an acceptable results at any station. As a result, the impact meteorological variables such as temperature and precipitation is undeniable. The use of these variables is necessary for improved prediction (Table 2).

The time series data, error, and standard deviation values for the selected scenarios for all data are presented in Fig. 8. Some models did not clearly detect peak values (Fig. 8a; Tapik station). As an example, ANN over-estimated the peak values in steps 150, 180, and 220. In step 35, the model under-estimated runoff. A similar tendency was observed for the ANN + WOA model, with delayed estimates. However, the ANN + PSO peak estimation was improved.

At the Band station, monthly runoff variation is larger compared to the previous station (Fig. 8b). Unlike the Tapik station, the ANN + WOA performed well for this station, especially in estimating the base discharge. On the other hand, the ANN + RSA often estimated the streamflow with some delay. However, the model worked well in estimating the peak values. The standard deviation of the error for this station is within the range of 1.3–2.05 MCM/month, and the error for all models follows a normal distribution (Fig. 8b). Similar results were obtained for the Babaroud station (Fig. 8c). For this station, the performance of the ANN + PSO model was best, where peak values were well estimated together with base flow. The standard deviation of the errors was the lowest among the models, being about 4.12 MCM/month. The error for all four stations followed a normal distribution (Fig. 8c). For Nazlo station it is indicated that the ANN + PSO hybrid model has acceptable performance in estimating streamflow (Fig. 8d). The results show that almost all models identified the base runoff well. Therefore, it can be deduced that for accurate estimation of the peaks, more information than used in this study might be required. The errors show that they all follow the normal distribution. The standard deviation of the errors and the average error for ANN + PSO were the lowest among the models (3.45 and − 0.528 MCM/month, respectively). ANN + WOA exerted the highest error standard deviation, equal to 11.5 MCM/month (Fig. 8d).

**Fig. 9:**
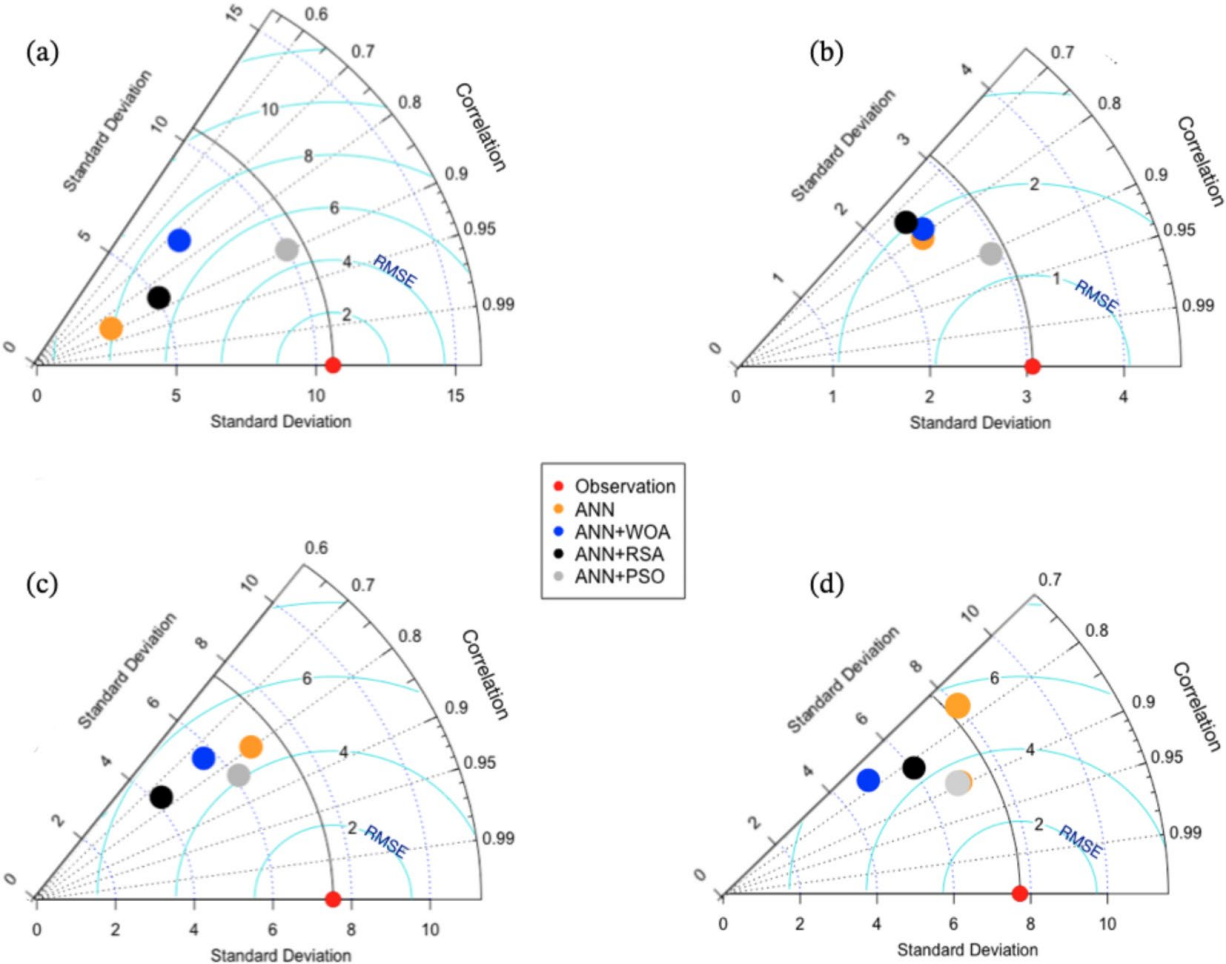
Taylor’s diagram: comparison between river flow forecasting models (**a**) Tapik station, (**b**) Band station, (**c**) Babaroud station, and (**d**) Nazlo station.

Figure 9 shows the Taylor diagram for the observational test data and the output of the employed models under the selected scenarios for each model. According to the figure, we compared the error rate, correlation, and standard deviation of the model outputs with observations. The ANN + PSO model has a good position compared to other models for the Tapik station (Fig. 9a). The correlation for this model was 0.90. The standard deviation was slightly larger than 10.0 MCM. The ANN + PSO is the closest to observations. The Fig. 9b (Band station), the correlation from the models ranges from 0.70 to 0.90. ANN + PSO model has the highest correlation coefficient (0.90) and ANN + RSA the lowest correlation (0.74). ANN + PSO hybrid model has the strongest performance among all models used in the present study. It is located close to the observational data in the diagram and has the lowest RMSE (ranging between 1 to 2.5 MCM/month). The two optimization algorithms RSA and WOA are located closer than ANN in the diagram. Similarly, for the Babaroud station, Fig. 9c shows that the correlation for the ANN + PSO model is better than other models (about 0.85, while those of the two models ANN + RSA and ANN are in the range of 0.80). For the Nazlo station (Fig. 9d), the correlations are mostly in the range of 0.75‒0.90. However, ANN and ANN + WOA show a weak performance in terms of estimation of baseflow.

**Fig. 10:**
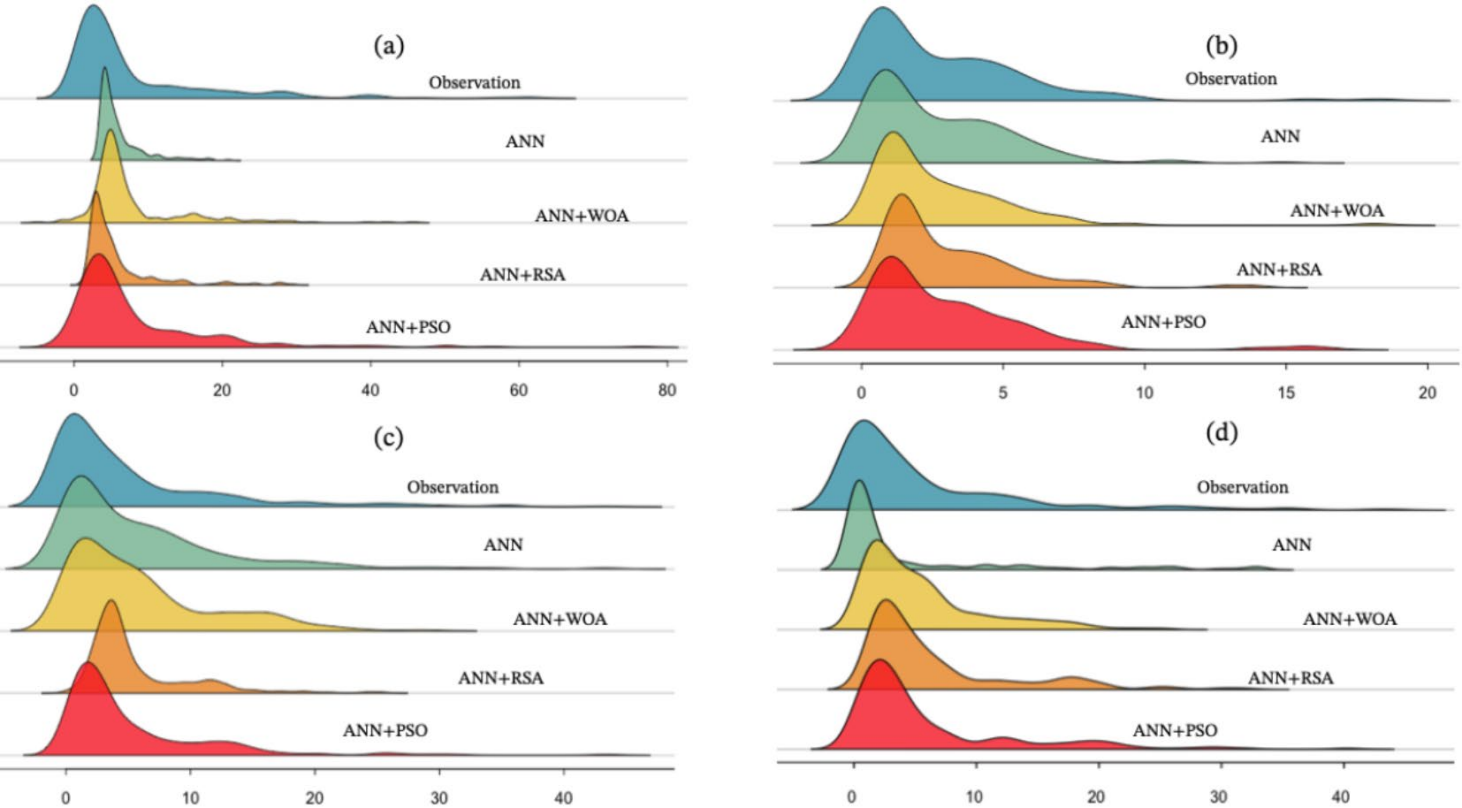
The Joyplot diagram shows how the estimated data by machine learning models compared to the observational reference data (**a**) Tapik station, (**b**) Band station, (**c**) Babaroud station, and (**d**) Nazlo station.

A ridgeline diagram was used to visualize the distribution of simulated data with machine learning models and observational data. In Fig. 10a for the Tapik station and the density curve part, there is a good overlap between the observational data and the two hybrid models (ANN + PSO and ANN + WOA), while the shape of the curve in the ANN model has a sharp peak. This means that the models estimated the base flow close to the observations. In contrast, in estimating the maximum values, the ANN + PSO model is close to observations. ANN under–estimates these values, and the ANN + PSO over-estimates them. For the Band station (Fig. 10b), there is not a good overlap between the hybrid models and observations. For Babaroud station (Fig. 10c), there is a good overlap in minimum values between the data of ANN + WOA models and observations. There is no significant overlap in estimating the values with data density that is high in that area, while the ANN + RSA and ANN + WOA models under–estimated the maximum values. For Nazlo station (Fig. 10d), ANN + PSO and ANN + RSA have a good overlap with each other, but are different from the observations and the other two models. For this station, the shape of ANN + PSO data distribution is very close to observational data.

**Fig. 11:**
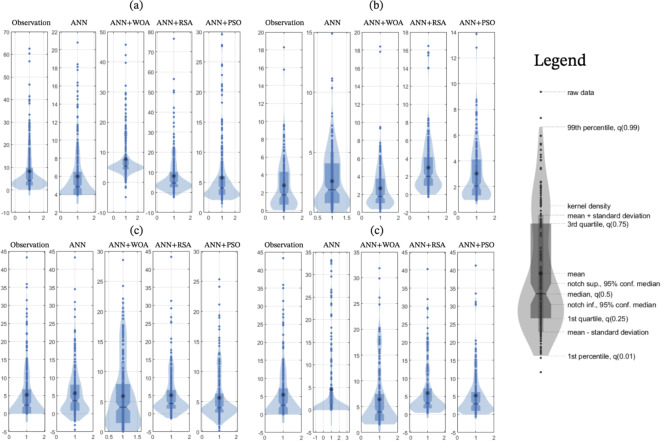
Violin diagrams showing how the estimated data by machine learning models compared to the observational reference data (**a**) Tapik station, (**b**) Band station, (**c**) Babaroud station, and (**d**) Nazlo station.

In Fig. 11, box plots and data density plots are presented in the form of a violin diagram. Based on Fig. 11a, for the Tapik station, the data distribution in the range of 0–20 (MCM) for ANN + PSO and ANN + WOA models is similar to observational data. Based on the boxplot for the Tapik station, the median of the ANN + PS model data is closer to the observed data. According to Fig. 11b, the shape of the data in the ANN + RSA and ANN + WOA models is different from the other two models and observational data. Also, the median of ANN and ANN + PSO data is consistent with observational data, but the outlier data of these two models is more than the outlier observation data. For Babaroud station (Fig. 11c), the four models used, the shape of the data of the ANN + WOA model is different from the other three models. For the Nazlo station (Fig. 11d), ANN and ANN + RSA models performed similarly. Although the shape of the data density in the ANN + PSO model is similar to the observational data, it over-estimated the base flow.

## Discussion

Accurate streamflow forecasting can help prevent disastrous phenomena such as floods and droughts. However, multiple variables impact river flow and add to the complexity of the problem. Often this is combined with data scarcity in developing countries. Consequently, the present study aimed to forecast river flow using a soft computing model in the face of data shortage. The non-hybrid ANN model used monthly temperature, precipitation, and river flow but did not perform satisfactorily. Similar results were found by Tongal and Booij^40^. They used the three variables temperature, precipitation, and evaporation for the purpose of river flow forecasting. This substantiates that ANN has a weaker performance in comparison with RF. The weaknesses of ANN stem from requiring more data, overfitting, and trapping the training algorithms in local minima^41^.

The results showed that by having only the discharge one month before, it is possible to predict the discharge using the ANN + RSA as shown by the NSE, RMSE, and MAE for the Band station. Overall, ANN + RSA had good estimation accuracy compared to other models. In addition, the performance of ANN + PSO was better than ANN + RSA and ANN + WOA, showing the high performance of this model in estimating streamflow. The results indicated that, in general, the performance of hybrid models was more appropriate than using single models. In rare cases, the performance of the single ANN model was somewhat similar to the hybrid models such as ANN + WOA. An example of this is the flow estimation at the Babaroud station. Hybrid models, however, do not always perform better than single models. In the evaluation of runoff estimation, five input scenarios were developed, in which the S5 scenario, which included all the input variables (runoff one and two months before, precipitation and temperature), provided best results for all models.

Evolutionary optimization algorithms can, however, improve the accuracy of non-hybrid models such as ANN and ANFIS in forecasting hydrological phenomena. Despite using only a few variables, ANN + PSO and ANN + RSA hybrid models performed with high accuracy in river flow forecasting, which is in line with the study by Azar et al. (2023)^21^. They found that HHO and PSO improve the results obtained from ANN. In comparison with the two other evolutionary algorithms, WOA was less effective in improving the accuracy of ANN. Compared to other studies, (e.g., Adnan et al.^36^ on the use of ANN + PSO and ANN + GWO models in streamflow estimation, Tikhamarine et al.^33^ on the use of ANN + GWO in streamflow prediction) it can be inferred that evolutionary algorithms can enhance the accuracy of streamflow prediction by neural networks. These finding aligns with the results of our study. However, it is important to acknowledge the limitations of our study, including the availability of long–term historical runoff data and the consideration of different time scales. The results indicated that the models’ performance varies based on the temporal and spatial scales of the data. Therefore, it is recommended that the aforementioned methods be assessed as well for daily and seasonal time intervals.

The results of the models varied for various stations. This shows the different performance of the models for different conditions. Therefore, selecting one model for all stations or different ranges is not recommended. It is necessary to use newer hybrid models and evaluate them for different ranges^42^. The results in this study showed that the models are not always accurate in estimating the peak values. It was not possible to properly estimate the peak values using the variables used in this research, and other variables are required for their proper estimation. These results are somewhat consistent with the research of Kisi et al.^43^. They found that some models (such as MARS or LSSVR) cannot accurately estimate peak values even with increasing number of variables and different lags. These models are suitable for estimating the monthly flow variation and base flow well, but they do not estimate the peak values very well^44^.

## Conclusions

We used ANN to forecast the streamflow of the main rivers in Urmia, northwest Iran and compared the obtained results with those of hybrid machine learning models. Given that machine learning models are highly accurate tools to forecast hydrological phenomena, especially in the case of data shortage, we aimed to forecast river flow using a minimum of observations. Consequently, monthly precipitation, temperature, and river flow of the previous month were used for the 2001‒2021 period. Five different input patterns were developed using various combinations of these three variables. Eventually, we compared the performance of the two models in terms of criteria such as NSE, MAE, and RMSE as well as time series diagrams. Even though ANN is popular among researchers as ANN + hydrid models, the findings of the present study show that in comparison with the hydrological model, ANN has a weak performance in forecasting river flow in some stations when facing data shortage. The non–hybrid ANN model has a weak performance especially at peak and trough points, possibly indicating that learning algorithms are trapped at local minima. To overcome the weaknesses of ANN (for tasting dataset), we developed hybrid models using the three evolutionary algorithms of WOA, RSA, and PSO and analyzed the results under different input scenarios. The results showed that the three proposed algorithms improved the performance of ANN to some extent. Among the three hybrid models, ANN + PSO demonstrated the most accurate results during both the training and testing phases. In general, the findings of this study corroborate that the proposed hybrid models are more accurate than non-hybrid models in predicting the peak and low points in the streamflow. Consequently, the evolutionary hybrid models developed in this study can be used as an efficient approach to gradually substitute non-hybrid artificial intelligence models in forecasting monthly streamflow. Moreover, the hybrid models presented in this paper can be adopted in future studies that focus on modeling other hydrological phenomena such as rainfall, rainfall-runoff, and the like.

## Data Availability

Upon request, data can be sent by the corresponding author via email, if possible, on a limited basis.
